# Use of a 3D Floating Sphere Culture System to Maintain the Neural Crest-Related Properties of Human Dental Pulp Stem Cells

**DOI:** 10.3389/fphys.2018.00547

**Published:** 2018-05-28

**Authors:** Alessandra Pisciotta, Laura Bertoni, Massimo Riccio, Jonathan Mapelli, Albertino Bigiani, Marcella La Noce, Monia Orciani, Anto de Pol, Gianluca Carnevale

**Affiliations:** ^1^Department of Surgery, Medicine, Dentistry and Morphological Sciences with Interest in Transplant, Oncology and Regenerative Medicine, University of Modena and Reggio Emilia, Modena, Italy; ^2^Department of Biomedical, Metabolic and Neural Sciences, Center for Neuroscience and Neurotechnology, University of Modena and Reggio Emilia, Modena, Italy; ^3^Department of Experimental Medicine, Unit of Biotechnologies, Medical Histology and Molecular Biology, Università degli Studi della Campania “Luigi Vanvitelli”, Naples, Italy; ^4^Department of Clinical and Molecular Sciences, Polytechnic University of Marche, Ancona, Italy

**Keywords:** 3D sphere culture system, neuro-ectomesenchyme, human dental pulp stem cells, neural crest, neuronal differentiation, immunomodulatory properties, CD34, Kir4.1

## Abstract

Human dental pulp is considered an interesting source of adult stem cells, due to the low-invasive isolation procedures, high content of stem cells and its peculiar embryological origin from neural crest. Based on our previous findings, a dental pulp stem cells sub-population, enriched for the expression of STRO-1, c-Kit, and CD34, showed a higher neural commitment. However, their biological properties were compromised when cells were cultured in adherent standard conditions. The aim of this study was to evaluate the ability of three dimensional floating spheres to preserve embryological and biological properties of this sub-population. In addition, the expression of the inwardly rectifying potassium channel Kir4.1, Fas and FasL was investigated in 3D-sphere derived hDPSCs. Our data showed that 3D sphere-derived hDPSCs maintained their fibroblast-like morphology, preserved stemness markers expression and proliferative capability. The expression of neural crest markers and Kir4.1 was observed in undifferentiated hDPSCs, furthermore this culture system also preserved hDPSCs differentiation potential. The expression of Fas and FasL was observed in undifferentiated hDPSCs derived from sphere culture and, noteworthy, FasL was maintained even after the neurogenic commitment was reached, with a significantly higher expression compared to osteogenic and myogenic commitments. These data demonstrate that 3D sphere culture provides a favorable micro-environment for neural crest-derived hDPSCs to preserve their biological properties.

## Introduction

Human dental pulp, a soft connective tissue contained within the pulp chamber of the tooth, is considered an interesting source of adult stem cells, due to the low-invasive procedures required for cell isolation, high content of stem cells and its peculiar embryological origin from neural crest (Goldberg and Smith, 2004; D’Aquino et al., 2009; Tirino et al., 2012). Particularly, neural crest cells originate during the formation of neural tube, at the 3rd week of embryo development, then undergo an epithelial-mesenchymal transition (EMT) and migrate to different body compartments under the control of several positive and negative regulatory factors (Shyamala et al., 2015). Following migration, neural crest cells generate the majority of craniofacial tissues, including tooth, fat, muscle, bone and cartilage tissues, as well as cranial peripheral ganglia and nerves, among other cell types, such as melanocytes. The differentiation potential of neural crest cells toward several lineages allows to consider the neural crest as the fourth germ layer. Based on their location within dental pulp, they can be easily isolated and might therefore be applied to cell therapy approaches for the regeneration of craniofacial injuries (Trainor, 2010). As well documented in literature, due to the heterogeneous nature of human dental pulp stem cells, distinct markers were used to select different subsets of stem cells, with different related behaviors, therefore it would be desirable, for regenerative medicine purposes, to obtain immunophenotypically pure stem cell populations. It has been widely demonstrated that, under appropriate stimuli, hDPSCs are able to differentiate toward osteogenic, myogenic and adipogenic lineages, islet like insulin producing β-cells, melanocytes, Schwann cells and neuronal lineage (Paino et al., 2010; Pisciotta et al., 2012, 2015a; Carnevale et al., 2013, 2016; Aurrekoetxea et al., 2015).

In particular, recent findings from our group highlighted the existence of two distinct subpopulations residing within the human dental pulp. We demonstrated that the neural crest-related subpopulation of hDPSCs expressing STRO-1, c-Kit and CD34 surface antigens showed a higher ability to commit toward neuronal lineage, although standard culture conditions appeared to affect stemness markers expression, proliferation rate and cell senescence at later passages (Pisciotta et al., 2015a). Previous findings showed that neural stem cells reside in niches owning properties that promote proliferation and prevent apoptosis of neural stem/progenitor cells (Studer et al., 2000), otherwise, when kept in adherent culture, stem cells could be directed to commit toward mesenchymal lineages (Mendez-Ferrer et al., 2010). It is well known that potassium channels could influence cell growth, maturation and differentiation and their expression was widely investigated in neural stem cells (Prüss et al., 2011). Particularly, Kir4.1 represents an inwardly rectifying K^+^ channel expressed in neural progenitor cells and in satellite glial cells surrounding primary afferent neurons in trigeminal ganglia. Nevertheless, its expression has never been investigated in hDPSCs so far.

Given all these considerations, the first aim of our study was to evaluate the ability of 3D sphere floating culture system to preserve the biological properties of hDPSCs immune-selected and enriched for STRO-1, c-Kit and CD34. Then, the study aimed to evaluate the expression of Kir4.1 in hDPSCs and the ability of these stem cells to express Fas and FasL, key molecules for the modulation of immune response. As widely reported in literature, FasL is expressed in stem cells and activated lymphocytess, as well as in cells residing in “immune-privileged” sites, such as the testis, the eye, and the nervous system (Brunlid et al., 2007). As a matter of fact, stemness maintenance associated to the expression of FasL would represent a meaningful goal in regenerative medicine. Therefore, it would be helpful to understand how FasL expression is modulated during stem cells differentiation.

## Materials and Methods

### Isolation and Immune Selection of hDPSCs

This study was carried out in accordance with the recommendations of Comitato Etico Provinciale – Azienda Ospedaliero-Universitaria di Modena (Modena, Italy), which provided the approval of the protocol (ref. number 3299/CE). Human DPSCs were isolated from third molars of adult subjects (*n* = 3; 18–25 years). All subjects gave written informed consent in accordance with the Declaration of Helsinki.

Cells were isolated from dental pulp as previously described (Pisciotta et al., 2012). Briefly, dental pulp was harvested from the teeth and underwent enzymatic digestion by using a digestive solution, consisting in 3 mg/mL type I collagenase plus 4 mg/mL dispase in α-MEM. Pulp was then filtered onto 100 μm Falcon Cell Strainers, in order to obtain a cell suspension. Cell suspension was then plated in 25 cm^2^ culture flasks and expanded in culture medium [α-MEM supplemented with 10% heat inactivated fetal bovine serum (FBS), 2 mM L-glutamine, 100 U/mL penicillin, 100 μg/mL streptomycin] at 37°C and 5% CO_2_. Following cell expansion, human DPSCs underwent magnetic cell sorting through MACS^®^ separation kit. Three subsequent immune-selections were performed by using primary antibodies: mouse IgM anti-STRO-1, rabbit IgG anti-c-Kit (Santa Cruz) and mouse IgG anti-CD34 (Chemicon-Millipore). The following magnetically labeled secondary antibodies were used: anti-mouse IgM, anti-rabbit IgG and anti-mouse IgG (Miltenyi Biotec). Firstly, cell suspension was selected by using anti-STRO-1 antibody. STRO-1^+^ hDPSCs were expanded and then selected by using anti-c-Kit antibody to obtain a STRO1^+^/c-Kit^+^ population. Likewise, the STRO-1^+^/c-Kit^+^ population was selected by anti-CD34 antibody to obtain the STRO-1^+^/c-Kit^+^/CD34^+^ hDPSCs population.

### Three-Dimensional Floating Sphere Culture System

For the generation of 3D floating spheres, STRO-1^+^/c-Kit^+^/CD34^+^ hDPSCs were seeded at a cell density of 3 × 10^3^ cells/cm^2^ in Ultra-Low attachment culture dishes (Corning) in serum-free DMEM/F12 culture medium (Euroclone) supplemented with 2 mM L-glutamine, 100 U/mL penicillin, 100 μg/mL streptomycin, 2% B27 supplement (Thermo Fisher Scientific), 20 ng/mL EGF (PeproTech), and 20 ng/mL b-FGF (PeproTech). Floating spheres were maintained and re-suspended in fresh culture medium up to passage 8. Through the whole culture time, it was carefully monitored that spheres did not exceed 250 μm in diameter and still appeared semi-transparent, to ascertain their viability (Gervois et al., 2015).

### Stemness and Neural Crest Markers Expression

The expression of the stemness markers STRO-1, c-Kit, CD34 was evaluated in freshly immunomagnetically selected hDPSCs and in sphere derived hDPSCs, by immunofluorescence analysis. Furthermore, after culturing hDPSCs through 3D sphere system, stem cells expanded for a prolonged time were assessed for the expression of the neural crest markers nestin, CD271 and SOX-10, as previously described (Carnevale et al., 2016). Cells were fixed in 4% paraformaldehyde in pH 7.4 phosphate buffer saline (PBS) for 20 min and washed in PBS. Samples were then blocked with 3% BSA in PBS for 30 min at room temperature and incubated with the primary antibodies [mouse IgM anti-STRO-1, rabbit anti-c-Kit (Santa Cruz Biotechnology), mouse anti-CD34 (Millipore), mouse anti-CD271(BioLegend), mouse anti-nestin (Millipore) and rabbit anti-SOX-10 (Abcam)] diluted 1:50 in PBS containing 3% BSA, for 1 h at room temperature. After washing in PBS containing 3% BSA, the samples were incubated for 1 h at room temperature with the secondary antibodies diluted 1:200 in PBS containing 3% BSA (goat anti-mouse Alexa647, goat anti-rabbit Alexa488, donkey anti-mouse Alexa546, goat anti-mouse Alexa488; Life Technologies). After washings with PBS, cells nuclei were stained with 1 μg/ml DAPI in PBS for 1 min, then samples were mounted with anti-fading medium (FluoroMount, Sigma-Aldrich). Samples not incubated with the primary antibody were used as negative controls. The multi-labeling immunofluorescence analyses were carried out avoiding cross-reactions between primary and secondary antibodies. Confocal imaging was performed by a Nikon A1 confocal laser scanning microscope as previously described (Pisciotta et al., 2015b). Confocal serial sections were processed with ImageJ software in order to obtain three-dimensional projections and image rendering was performed by Adobe Photoshop Software. Counting of cells positively labeled against stemness and neural crest markers was performed on 10 randomly chosen fields on three different slides in double-blind manner.

### Cell Proliferation

The proliferation rate was determined on STRO-1^+^/c-Kit^+^/CD34^+^ hDPSCs seeded at passage 6 at the density of 2 × 10^3^ cells/cm^2^ and cultured for 1 week in DMEM/F12 culture medium (Euroclone) supplemented with 2% FBS, 2 mM L-glutamine, 100 U/mL penicillin, 100 μg/mL streptomycin, 20 ng/mL epidermal growth factor (EGF, PeproTech), and 20 ng/mL basic fibroblast growth factor (b-FGF, PeproTech) until confluence was reached. Each day cells were counted; the mean of cell number was calculated on three experimental samples for each time point and cell density was expressed as mean of cells/cm^2^ ± standard deviation (SD). The population doubling time (PDT) was calculated in the phase of exponential growth by the following formula (Pisciotta et al., 2015a).

$$\begin{array}{c} PDT=\;\frac{\log_{10}\left(2\right)\;\times \;\Delta T}{\log_{10}(N_{7d})-\;\log_{10}(N_{1d})\;} \end{array}$$

*N*_7d_ is the cell number at day 7 and *N*_1d_ is the cell number at day 1.

### Cell Senescence and Apoptosis

In order to test the occurrence of cell senescence STRO-1^+^/c-Kit^+^/CD34^+^ hDPSCs cultured in expansion medium (DMEM/F12 plus 2% FBS, 2 mM L-glutamine,100 U/mL penicillin, 100 μg/mL streptomycin, 20 ng/mL EGF and 20 ng/mL b-FGF) up to late passages (P6), were assayed for β-galactosidase activity, as formerly described (Carnevale et al., 2013). Three samples were analyzed for each culture condition and the percentage of senescent cells was calculated.

Furthermore, Western blot analysis was performed to evaluate the apoptosis process by assessing the expression of cleaved caspase 3.

### Multilineage Differentiation of hDPSCs

Human DPSCs obtained after disaggregation of floating 3D spheres were seeded at cell densities of 3 × 10^3^ cells/cm^2^ and 4 × 10^3^ cells/cm^2^, respectively, for the induction toward osteogenic and myogenic lineages. Osteogenic differentiation of hDPSCs was induced for 3 weeks as previously described (Bianchi et al., 2017; Paino et al., 2017). Cells were cultured in osteogenic medium consisting in α-MEM supplemented with 5% FBS, 100 μM 2P-ascorbic acid, 100 nM dexamethasone, and 10 mM β-glycerophosphate, for 2 weeks. For myogenic induction, hDPSCs were preliminarily treated with the demethylating agent 5-aza-2′-deoxycytidine (10 μM) for 24 h, then differentiation was carried out in DMEM Low Glucose (Euroclone, Italy) plus 5% horse serum, 0.5% chicken serum, 2 mM L-glutamine, 100 U/ml penicillin, 100 μg/ml streptomycin, and 10 nM insulin for 3 weeks, as formerly described (Pisciotta et al., 2015b). At the end of the induction period, confocal immunofluorescence analysis was performed to evaluate the achievement of both the commitments, through the expression of specific tissue related markers. The following primary antibodies were used: mouse anti-osteopontin (OPN), rabbit anti-Runx-2 (Abcam) and rabbit anti-desmin (Sigma-Aldrich).

### Neurogenic Differentiation of hDPSCs

For the induction of neurogenic commitment, hDPSCs cultured in floating spheres, as described above, were collected and disaggregated. About 4 × 10^3^ cells/cm^2^ were seeded on glass coverslips, formerly treated with 0.005% poly-L-lysine in 6-well culture plates. Neurogenic differentiation was induced with serum-free DMEM/F12 medium supplemented with 2 mM L-glutamine, 100 U/mL penicillin, 100 μg/mL streptomycin, 2% B27 supplement (Thermo Fisher Scientific), 20 ng/mL EGF, 20 ng/mL b-FGF, N2 supplement (Thermo Fisher Scientific), 100 ng/mL human nerve growth factor (hNGF, PeproTech), 10 ng/mL human brain derived neurotrophic factor (hBDNF, PeproTech) and 0.5 μM all-transretinoic acid (RA, Sigma-Aldrich). Neurogenic induction was performed for 3 weeks and differentiation medium was changed twice a week. At the end of the induction period, hDPSCs were assayed for the expression of neuronal differentiation markers by immunofluorescence analysis, as described above, using the following primary antibodies: mouse anti-MAP-2 (Sigma-Aldrich), rabbit anti-β-III-Tubulin (Cell Signaling). To further investigate the effective induction toward neuronal commitment, a comparative evaluation of nestin/β-III-Tubulin expression was performed between undifferentiated hDPSCs and differentiated hDPSCs, by immunofluorescence analysis. Differential expression of nestin underwent semi-quantitative measurement through pseudocolor analysis, in particular, blue to white arrays the colors in a spectrum with blue assigned to a lower value than white.

Moreover, Western blot analysis was carried out to evaluate the induction of neurogenic differentiation, through the expression of β-III-Tubulin.

### Intracellular Electrophysiology: Patch Clamp Analysis

Intracellular recordings were performed by positioning neuronal differentiated hDPSCs in a recording chamber under the objective of an upright epifluorescence microscope (Zeiss, Axioscope). Cells were continuously perfused with extracellular fresh Tyrode’s solution containing 140 mM NaCl,4 mM KCl, 2 mM CaCl_2_, 2 mM MgCl_2_, 10 mM Glucose, 10 mM Hepes. pH was adjusted to 7.4 with NaOH. Whole-cell recordings from differentiated hDPSCs were obtained with patch-clamp technique by using an Axopatch 200B amplifier (Molecular Devices, Union City, CA, United States). Patch pipettes were filled with a solution consisting in 126 mM *K*-gluconate, 8 mM NaCl, 15 mM glucose, 5 mM HEPES, 1 mM MgSO_4_, 0.1 mM BAPTA-4K, 0.05 mM BAPTA-Ca^2+^, 3 mM ATP, 100 μM GTP; pH was adjusted to 7.2 with KOH. This solution maintained resting free-[Ca^2+^] at 100 nM and pipettes had a resistance of 3–5 MΛ before seal formation. In Voltage clamp configuration, current relaxation induced by a 10 mV step from the holding potential of -70 mV was analyzed to ensure recordings stability. Voltage-clamp recordings were performed at room temperature (25.5°C) in Tyrode’s solution. Data were recorded with an Axopatch 200B amplifier, digitized with a Digidata 1200 interface (500 μs/point) and analyzed with Pclamp software (Axon Instruments, Foster City, CA, United States). In voltage clamp recordings, leak subtraction was performed by using a P4 protocol. In order to quantify currents kinetics, we calculated the time to reach the peak (time-to-peak) for inward transient currents. All data were reported as mean ± SEM.

### Expression of Kir4.1, Fas and FasL

The evaluation of inwardly rectifying K^+^ channel Kir4.1 was performed through Western Blot and immunofluorescence analyses in undifferentiated 3D-derived hDPSCs and in hDPSCs committed toward neuronal lineage, after 3 weeks of induction.

At the same time, the expression of Fas and FasL was evaluated by Western Blot and immunofluorescence analyses in undifferentiated STRO-1^+^/c-Kit^+^/CD34^+^ hDPSCs. Particularly, the expression of Fas and FasL was investigated by Western blot analysis on hDPSCs population after culture in sphere system and after adherent standard culture conditions, respectively, at early passages. Moreover, after 3 weeks of neuronal induction the expression of FasL was also investigated and compared with STRO-1^+^/c-Kit^+^/CD34^+^ hDPSCs differentiated toward osteogenic and myogenic lineages by Western Blot and immunofluorescence analyses. Immunofluorescence analysis was carried out as described above by using the following primary antibodies: rabbit anti-Kir4.1 (1:100; StressMarq Biosciences), rabbit anti-FasL (1:100; Cell Signaling). Then the primary antibodies were revealed by a goat anti-rabbit Alexa488 secondary antibody, diluted 1:200. Nuclei were counterstained with 1 μg/ml DAPI in PBS.

### Western Blot Analysis

Whole cell lysates were obtained as previously described (Pisciotta et al., 2012). Thirty μg of protein extract for each sample were quantified by a Bradford Protein Assay (Bio-Rad) and underwent SDS-polyacrylamide gel electrophoresis and were then transferred to PVDF membranes. The following antibodies were used: rabbit anti-cleaved caspase 3, rabbit anti-β III Tubulin, rabbit anti-FasL, mouse anti-Fas (Cell Signaling) and rabbit anti-Kir4.1 (StressMarq Biosciences) diluted 1:1000 in Tris buffered saline Tween 20 + 2% BSA and 3% non-fat milk. Membranes were then incubated with HRP-conjugated anti-rabbit secondary antibodies diluted 1:3000, for 30 min at room temperature. The membranes were visualized using ECL (enhanced chemiluminescence, Amersham, United Kingdom). Anti-actin antibody was used as a control of protein loading. Densitometry was performed by Fiji ImageJ software. An equal area was selected inside each band and the mean of gray levels (in a 0–256 scale) was calculated. Data were then normalized to values of background and of control actin band (Pisciotta et al., 2012).

### Statistical Analysis

All experiments were performed in triplicate. Data were expressed as mean ± standard deviation (SD). Differences between two experimental conditions were analyzed by paired, Student’s *t*-test. Differences among three or more experimental samples were analyzed by ANOVA followed by Newman–Keuls *post hoc* test (GraphPad Prism Software version 5 Inc., San Diego, CA, United States). In any case, significance was set at *P* < 0.05.

## Results

### Evaluation of Stemness Markers and 3D Sphere Generation

**FIGURE 1:**
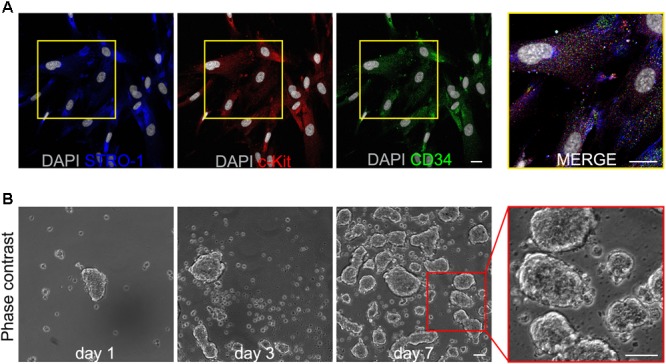
**(A)** Immunofluorescence analysis on immunomagnetically selected hDPSCs shows the expression of the stemness markers c-Kit, STRO-1 and CD34. Bar: 10 μm. **(B)** Phase contrast images demonstrate the ability of selected hDPSCs to aggregate in floating three-dimensional spheres, as early as 24 h after cell seeding, and to grow in size through culture time, up to passage 8. On the right, a higher magnification emphasizing the semi-transparent appearance of 3D spheres. Bar: 100 μm.

After isolation and expansion, immunomagnetically selected human DPSCs were evaluated for the expression of the three stemness markers STRO-1, c-Kit and CD34, by immunofluorescence analysis. As shown in **Figure 1A**, almost all hDPSCs (∼97%) effectively expressed all the three markers, confirming the selection of a pure hDPSCs subpopulation.

**FIGURE 2:**
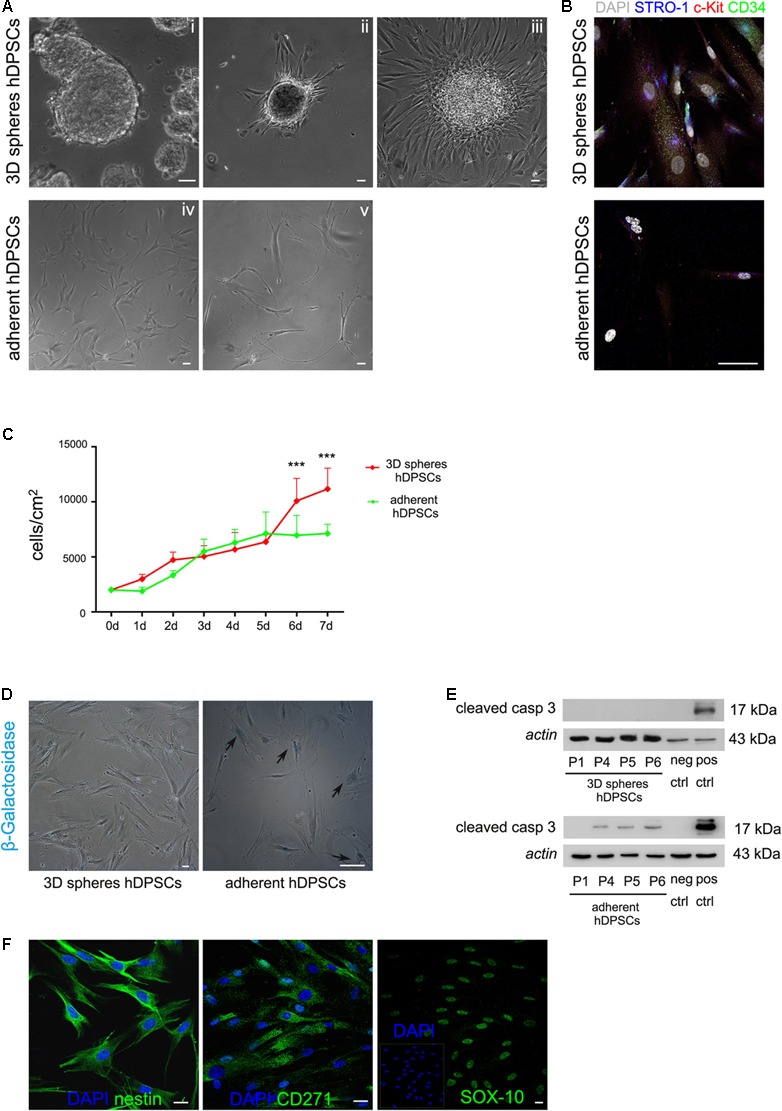
**(A)** Phase contrast images showing that hDPSCs, being cultured for repeated passages as floating three-dimensional spheres (i), attached to glass coverslips formerly treated with 0.005% poly-L-lysine (ii) and started to spread out of the spheres (iii), also keeping their fibroblast-like morphology. Human DPSCs cultured under adherent conditions showed a shift in cell morphology from the initial seeding (iv) up to passage 8 (v). **(B)** Immunofluorescence analysis showed the expression of the stemness markers c-Kit, STRO-1, and CD34 in hDPSCs derived from 3D sphere culture and in hDPSCs cultured in adherent conditions, following prolonged culture times. Bar: 50 μm. **(C)** Evaluation of proliferation ability in STRO-1^+^/c-Kit^+^/CD34^+^ hDPSCs cultured for 1 week, after 3D sphere culture and adherent culture conditions, respectively. Values were expressed as mean ± SD (^∗∗∗^*P* < 0.001 3D spheres hDPSCs vs. adherent hDPSCs). **(D)** Cell senescence was evaluated through the assay of β-galactosidase activity in 3D sphere-derived STRO-1^+^/c-Kit^+^/CD34^+^ hDPSCs and STRO-1^+^/c-Kit^+^/CD34^+^ hDPSCs cultured in adherent conditions, respectively, for 6 passages. Blue staining indicates cells positive for β-galactosidase activity (black arrows). Bar: 50 μm. **(E)** Western blot analysis of cleaved caspase 3 expression by STRO-1^+^/c-Kit^+^/CD34^+^ hDPSCs at different passages, following culture as 3D spheres and adherent culture conditions, respectively. Negative and positive controls of cleaved caspase 3 (Cell Signaling) were loaded on the right. Actin bands were presented as control of protein loading. **(F)** Immunofluorescence analysis was performed on STRO-1^+^/c-Kit^+^/CD34^+^ hDPSCs derived from 3D sphere culture, at late passages, to assess the expression of neural crest markers nestin, CD271 and SOX-10. Nuclei were counterstained with DAPI. Bar: 10 μm.

Following further expansion, hDPSCs were cultured in 3D sphere culture medium, starting from a cell seeding density of 3 × 10^3^ cells/cm^2^. Under these culture conditions hDPSCs revealed the ability to aggregate in floating three-dimensional structures, the 3D spheres, as early as 24 h after cell seeding (**Figure 1B**). Through the culture time spheres increased in number and size, thus, in order to avoid that growing spheres lose their semi-transparent appearance and undergo cell death, they were harvested and centrifuged, then were resuspended in fresh sphere culture medium and maintained up to passage 8 (**Figure 1B**). At this time point, floating spheres were collected and plated to attach on glass coverslips, previously treated with 0.005% poly-L-lysine (**Figures 2Ai,ii**). Following adhesion, hDPSCs started to grow out of the spheres and, as time passed by, gradually proliferated, as shown in phase contrast images (**Figure 2Aiii**). The counterpart STRO-1^+^/c-Kit^+^/CD34^+^ hDPSCs cultured in standard adherent conditions in α-MEM plus 10% FBS, 2 mM L-glutamine,100 U/mL penicillin, and 100 μg/mL streptomycin, revealed a change in morphology from initial seeding up to passage 8 (**Figures 2Aiv,v**). Then, sphere derived hDPSCs revealed that the expression of STRO-1, c-Kit and CD34 markers was maintained, following the use of sphere culture system for multiple passages whereas a notably reduced expression of the three stemness markers at passage 8 was observed in hDPSCs maintained in standard adherent conditions (**Figure 2B**).

### Cell Proliferation, Cell Senescence, Apoptosis and Neural Crest Markers

In order to investigate the growth kinetics of STRO-1^+^/c-Kit^+^/CD34^+^ hDPSCs derived from 3D sphere culture, proliferation rate was measured on cells seeded at 2 × 10^3^ cells/cm^2^ and maintained for 7 days in expansion medium consisting of DMEM/F12 culture medium supplemented with 2% FBS, 2 mM L-glutamine, 100 U/mL penicillin, 100 μg/mL streptomycin, 20 ng/mL EGF, and 20 ng/mL b-FGF. The counterpart STRO-1^+^/c-Kit^+^/CD34^+^ hDPSCs cultured in adherent conditions in α-MEM plus 10% FBS, 2 mM L-glutamine,100 U/mL penicillin, and 100 μg/mL streptomycin were taken as control. Each day cell count was performed on ten randomly chosen fields of 1.25 mm^2^. As shown in **Figure 2C**, cells demonstrated a steadily rising growth with a more evident increase from day 5 up to day 7 and a resulting population doubling time (PDT) of ∼21.8 ± 3.9 h. Moreover, cell senescence, assessed by the evaluation of β-galactosidase activity at passage 6, revealed that, under these culture conditions, STRO-1^+^/c-Kit^+^/CD34^+^ hDPSCs did not show any significant senescence by microscopic observation, as reported in **Figure 2D**. On the other hand, when STRO-1^+^/c-Kit^+^/CD34^+^ hDPSCs were cultured in adherent conditions a statistically significant decrease in proliferation rate was detected at days 6 and 7 (^∗∗∗^*P* < 0.001 adherent hDPSCs vs. 3D spheres hDPSCs) and the PDT was ∼35.2 ± 5.8 h; moreover, cells barely reached confluence and revealed a high percentage of senescent cells at passage 6 (**Figure 2D**, arrowheads; ∼85% adherent hDPSCs vs. ∼1.5% 3D spheres hDPSCs). Western blot analysis did not show any expression of cleaved caspase 3 in STRO-1^+^/c-Kit^+^/CD34^+^ hDPSCs from 3D sphere culture at different cell passages, with respect to hDPSCs cultured in adherent conditions (**Figure 2E**). Besides confirming the maintenance of cell proliferation without the occurrence of significant cell senescence or apoptosis at late passages, almost all 3D sphere-derived hDPSCs sub-population (∼95%) stained positive for the expression of neural crest markers nestin, CD271 and SOX-10 (**Figure 2F**), as demonstrated by immunofluorescence analysis.

### hDPSCs Derived From 3D Sphere Culture Differentiate Toward Osteogenic and Myogenic Lineages

**FIGURE 3:**
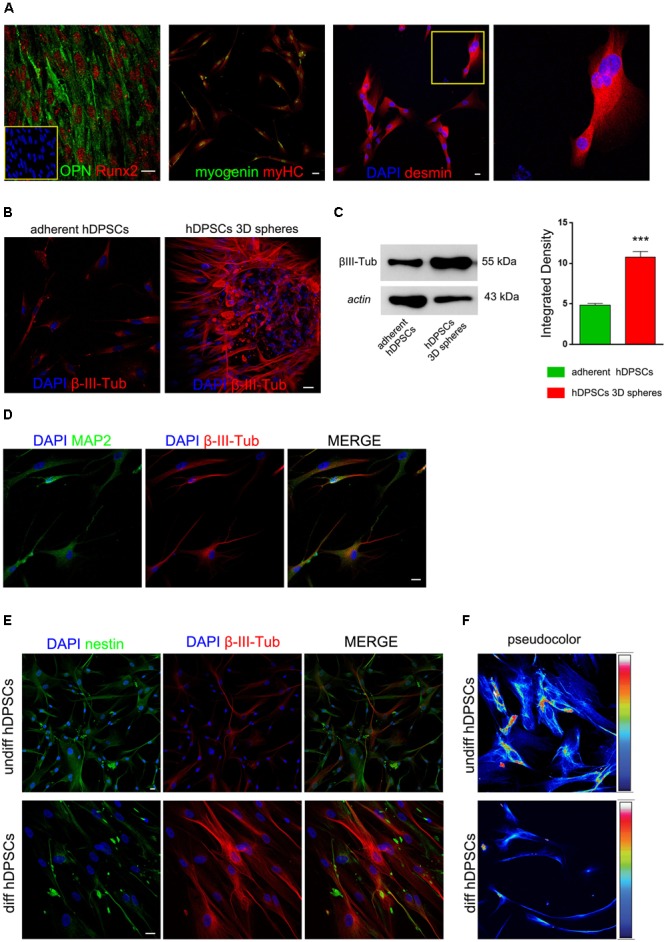
Commitment of hDPSCs toward osteogenic and myogenic lineages was evaluated by immunofluorescence analysis **(A)**. On the left, positive labeling against bone related markers OPN and Runx2; on the right, positive labeling against the myogenic markers myogenin, myosin heavy chain (myHC) and desmin. Multi-nucleated syncytia are detectable among desmin positive cells and are reported in the insert at higher magnification. Bar: 10 μm. Evaluation of β-III-Tubulin expression was carried out by immunofluorescence **(B)** and Western blot **(C)** analyses in undifferentiated STRO-1^+^/c-Kit^+^/CD34^+^ hDPSCs, following adherent (left) and sphere culture conditions (right), respectively. Western blot analysis confirmed the expression of β-III-Tubulin in undifferentiated STRO-1^+^/c-Kit^+^/CD34^+^ hDPSCs cultured either in adherent and sphere culture conditions. Actin bands were presented as control of protein loading. Densitometric analysis revealed a statistically significant higher expression of β-III-Tubulin in hDPSCs cultured as floating spheres (^∗∗∗^*P* < 0.001 vs. adherent hDPSCs). **(D)** Neuronal differentiation of hDPSCs was investigated, after 3 weeks of induction, by immunofluorescence analysis of MAP-2 and β-III-Tubulin. **(E)** Immunofluorescence analysis demonstrated a decrease in nestin expression and, at the same time, a stronger expression of β-III-Tubulin, as hDPSCs proceeded toward neuronal commitment. **(F)** Pseudocolor images provided semi-quantitative measurement of nestin expression in undifferentiated and differentiated hDPSCs. Bar: 10 μm.

The multipotency of hDPSCs derived from sphere culture system was evaluated after inducing the commitment toward osteogenic and myogenic lineages. After 3 weeks of induction, hDPSCs showed the expression of typical osteogenic markers, i.e., OPN and Runx-2, as demonstrated by immunofluorescence confocal analysis (**Figure 3A**). Similarly, hDPSCs were also able to achieve the myogenic commitment, as demonstrated by the positive labeling against the myogenic markers myogenin, myosin heavy chain and desmin. Moreover, myotube formation was detected, as demonstrated by the presence of multi-nucleated syncytia (**Figure 3A**).

### Evaluation of Neuronal Commitment of hDPSCs

As shown in **Figure 3**, the expression of β-III-Tubulin was investigated in undifferentiated hDPSCs following adherent and 3D sphere culture conditions, respectively. Particularly, β-III-Tubulin was detected in undifferentiated hDPSCs when maintained in either adherent or floating sphere culture conditions, however, a statistically significant higher expression was revealed in sphere culture system, as shown by immunofluorescence analysis (**Figure 3B**) and further confirmed by densitometry performed after Western blot analysis (**Figure 3C**; ^∗∗∗^*P* < 0.001 vs. adherent hDPSCs). After 3 weeks of induction by culturing hDPSCs in differentiation medium, neuronal commitment was investigated by immunofluorescence analysis of MAP-2 and β-III-Tubulin, which revealed an effective commitment of hDPSCs toward the neuronal lineage (**Figure 3D**). Moreover, as the induction of hDPSCs toward neuronal commitment proceeded, a reduced expression of nestin was observed in parallel with an increased expression of β-III-Tubulin, as shown in **Figure 3E**. Particularly, this data was confirmed by pseudocolor analysis performed on undifferentiated hDPSCs and on hDPSCs committed toward neuronal lineage (**Figure 3F**).

### Electrophysiological Recordings

**FIGURE 4:**
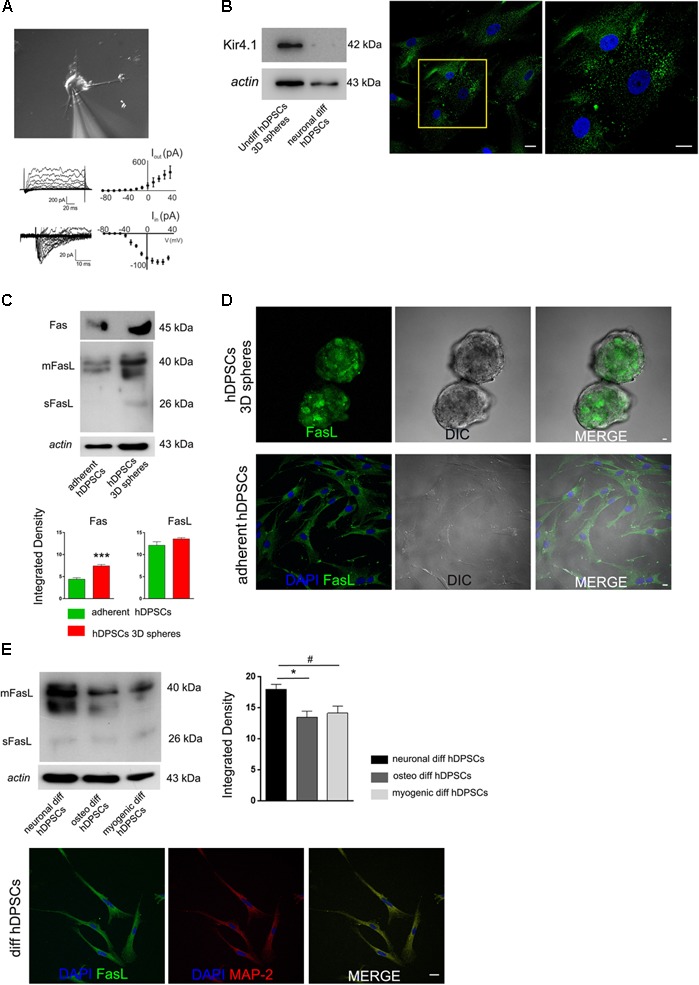
**(A)** Intracellular patch-clamp recordings performed on neuronal differentiated hDPSCs. Cells exhibit large outward (*I*_out_) and tiny inward transient currents (*I*_in_). Inward transient currents showing a small amount of currents at the peak of activation and kinetics compatible with transient sodium currents. Outward currents displaying larger values at the peak and kinetics compatible with currents produced by voltage-dependent potassium channels, responsible of action potential repolarization. **(B)** Western blot analysis (left) of Kir4.1 in undifferentiated 3D-derived hDPSCs and in hDPSCs following neuronal commitment. Immunofluorescence analysis against Kir4.1 was performed on undifferentiated STRO-1^+^/c-Kit^+^/CD34^+^hDPSCs following 3D culture (right). High magnification is referred to yellow insert. **(C)** Western blot analysis of Fas and FasL, and **(D)** immunofluorescence analysis of FasL expression were carried out on undifferentiated hDPSCs, cultured either as floating spheres or under adherent conditions. Densitometry of Fas and FasL bands is shown at the bottom (^∗∗∗^*P* < 0.001, Fas expression in hDPSCs 3D spheres vs. adherent hDPSCs). No statistically significant differences were reported for FasL expression between the two culture conditions. **(E)** Western blot analysis of FasL performed on hDPSCs induced toward neuronal, osteogenic and myogenic lineages, respectively. Actin bands were presented as control of protein loading. Densitometric analysis revealed that FasL expression was higher following induction of neuronal commitment in hDPSCs, when compared to osteogenic and myogenic commitments (^∗^*P* < 0.05 vs. osteogenic diff hDPSCs, ^#^*P* < 0.05 vs. myogenic diff hDPSCs). Immunofluorescence analysis on differentiated hDPSCs showed FasL expression following induction to neuronal commitment, as demonstrated by positive immunolabeling against MAP-2 and FasL. Bar: 10 μm.

In order to assess the presence of functional ionic channels, intracellular patch-clamp recordings were performed on hDPSCs committed toward neuronal lineage after 3 weeks of induction (**Figure 4A**). In voltage clamp configuration, hDPSCs passive properties were monitored throughout recordings to ensure stability (Membrane capacitance 34.8 ± 5.4 pF, Input Resistance 530.2 ± 140.4 MΩ, Resting membrane potential -71 ± 4.8 mV; *n* = 4). When cells were injected with depolarizing current steps ranging from -80 to +40 mV, they exhibited large outward (*I*_out_) and tiny inward transient currents (*I*_in_), as shown in **Figure 4A**. Although, these latter showed a small amount of currents at the peak of activation (83.4 ± 4.5 pA at +30 mV; *n* = 4 cells; **Figure 4A**) and kinetics were compatible with transient sodium currents (time to peak 2.1 + 0.8 ms *n* = 4; **Figure 4A**). Conversely, outward currents exhibited larger values at the peak (726 ± 141 pA at +30 mV, *n* = 4; **Figure 4A**) and showed kinetics compatible with currents generated by voltage-dependent potassium channels responsible of action potential repolarization.

### Evaluation of Kir.4.1 and Fas/FasL in hDPSCs: How Culture System and Lineage Induction Can Modulate Their Expression

The expression of inwardly rectifying potassium channel Kir4.1 was investigated in undifferentiated STRO-1^+^/c-Kit^+^/CD34^+^ hDPSCs after 3D sphere culture and in hDPSCs committed toward neuronal lineage after 3 weeks of induction. Western blot analysis revealed that Kir4.1 was expressed in undifferentiated hDPSCs, whereas it was lost following the induction toward neuronal commitment (**Figure 4B**).

The expression of Fas and FasL, which play an important role in the regulation of immune system, was investigated in hDPSCs, either when kept in undifferentiated conditions and after 3 weeks of neuronal induction. As shown by Western blot analysis in **Figure 4C**, hDPSCs expressed Fas either when cultured in adherent conditions and in sphere system, although, a statistically significant increase was revealed after culturing in floating 3D spheres (^∗∗∗^*P* < 0.001 vs. adherent hDPSCs; **Figure 4C**). On the other hand, FasL was expressed by hDPSCs when cultured in both conditions without any statistically significant difference (**Figure 4C**). This data was confirmed by immunofluorescence analysis, as reported in **Figure 4D**.

The expression of FasL was also investigated and compared in hDPSCs following the induction toward neuronal, osteogenic and myogenic commitment. Interestingly, as shown in **Figure 4E**, FasL expression was still detected in hDPSCs after 3 weeks of neuronal induction, as demonstrated by Western blot and immunofluorescence analyses. Noteworthy, FasL expression was statistically significant higher when compared to hDPSCs induced toward osteogenic and myogenic lineages, respectively (^∗^*P* < 0.05 vs. osteogenic differentiation of hDPSCs and ^#^*P* < 0.05 vs. myogenic differentiation of hDPSCs).

## Discussion

Cell therapy approaches based on the use of neural crest derived stem cells, due to their embryological origin related properties, are highly promising for the regeneration of different craniofacial tissue injuries. Among the neural crest derived stem cells conserved in several regions of the adult body, human dental pulp represents an easily accessible source to isolate stem cells with low invasiveness procedures (Gilbert, 2000).

We recently described the existence of two distinct subpopulations of hDPSCs attributable to the peculiar embryological origin of dental pulp. The hDPSCs subpopulation expressing c-Kit, STRO-1, and CD34 showed a high tendency toward the neurogenic commitment. However, we recognized that culture and expansion conditions for such stem cells needed to be optimized in order to obtain a sufficient number of cells, allowing to maintain biological/stemness properties and cell proliferation, which are necessary criteria to provide a suitable source of progenitor cells for regenerative medicine purposes (Pisciotta et al., 2015a).

In light of the future potential application of this hDPSCs subpopulation after proper *in vivo* investigations, the focus of our present study was to develop optimal culture conditions for preserving their ectomesenchyme related properties and their differentiation potential. Based on these premises and the existing literature, we chose to culture hDPSCs expressing c-Kit, STRO-1, and CD34, as floating spheres, with the use of serum free expansion medium supplemented with b-FGF and EGF. Our data showed that hDPSCs were able to form three-dimensional spheroid structures, which kept growing and increasing in size. After prolonged culture in this 3D sphere system, hDPSCs still expressed the three stemness markers for which they were initially immunomagnetically selected. Moreover, following disaggregation of floating spheres and adhesion on tissue culture plates, in serum free and growth factors supplemented medium, hDPSCs preserved their proliferation ability, without the occurrence of significant cell senescence or cell apoptosis. Indeed, besides preserving their stemness hDPSCs also maintained their ability to differentiate toward osteogenic and myogenic lineages. Most likely the presence of several c-Kit^+^/STRO-1^+^/CD34^+^ hDPSCs positively stained for β-galactosidase activity might reveal that, when maintained in adherent culture till late passages, this stem cells sub-population lost stemness and proliferation ability, gradually proceeding toward cell senescence or cell differentiation. Furthermore, at the same time, a shift in cell morphology was observed when hDPSCs were maintained in adherent culture: particularly, cells lost their typical fibroblast-like morphology, becoming flattened. The preserved cell proliferation ability of hDPSCs cultured in 3D spheres could be associated to the maintenance of Fas receptor expression. As a matter of fact, Fas not only is a membrane receptor related to apoptotic pathway, but it is also involved in cell proliferation (Carnevale et al., 2017).

Noteworthy, 3D culture conditions proved to be effective for maintaining the expression of neural crest markers, i.e., nestin, CD271 and SOX-10, thus suggesting that microenvironment conditions within the spheres are favorable for the preservation of stemness and neuro-ectomesenchyme properties of these stem cells (Gervois et al., 2015). Moreover, hDPSCs cultured as floating spheres showed a strong expression of β-III-Tubulin, at late passages, which was significantly higher when compared to hDPSCs cultured in adherent conditions. The expression of β-III Tubulin has been suggested to be related to neural progenitor cells and neural crest stem cells, besides being one of the earliest markers to signal neuronal commitment. The progressive commitment toward neuronal lineage was also confirmed by the expression of MAP-2. Moreover, as neuronal differentiation proceeded, a decrease in nestin expression was observed, further confirming the achievement of the commitment. Taken together, our data are in accordance with several reports showing that a close connection/interaction between neural progenitor cells is primary for the achievement of neuronal commitment, which can be obtained through sphere culture system (Schlett et al., 2000; Tarnok et al., 2002).

Preliminary data obtained from electrophysiological recordings revealed that, besides the expression of late neuronal differentiation markers, hDPSCs exhibited functional ionic currents compatible with fast transient sodium and voltage-dependent potassium currents, typically expressed in neurons. It should be noted that current clamp recordings did not show the presence of action potential, which could be due to a small number of active channels, given the small values of peak currents. However, the analysis of current kinetics confirmed that neuronal committed hDPSCs were capable of generating ionic currents closely resembling sodium and potassium currents, which are fundamental to generate action potentials. Indeed, larger current values could be measured in cells recorded at later stages of terminal neuronal differentiation. In compliance with reports from several research groups, although sphere-derived hDPSCs were kept in appropriate differentiating conditions for 3 weeks, this induction period might be ineffective to achieve a full neuronal cell maturation (Zhang and Zhang, 2010; Espuny-Camacho et al., 2013). Biella et al. (2007) demonstrated a progressive increase in current amplitude during neuronal maturation and, ultimately, action potential firing.

The expression of Kir4.1 was widely investigated and demonstrated in neural progenitor/stem cells and in satellite glial cells surrounding primary afferent neurons in trigeminal ganglia. As shown in a previous study (Vit et al., 2008), Kir4.1 is thought to influence cell growth, differentiation and maturation. Particularly, high levels of Kir4.1 were observed in nestin positive cells and were maintained in glial cells, whereas neurons do not express Kir4.1. Our findings are in accordance with such evidence, indeed, undifferentiated hDPSCs being strongly positive for nestin also expressed high levels of Kir4.1. Conversely, the expression of Kir4.1 decreased in hDPSCs being progressively committing to neuronal lineage, together with a simultaneous reduction in nestin expression.

Additional studies, either by using pharmacological block of ionic currents or later times of differentiation will be necessary to further investigate the nature of the recorded currents.

Furthermore, the ability of stem cells to modulate the immune response through different mechanisms, including Fas/Fas ligand pathway, could be another important goal in regenerative medicine.

Our data demonstrated that hDPSCs cultured through sphere generation maintained high levels of FasL, similarly to hDPSCs kept in adherent culture. Furthermore, we observed that the expression of FasL was preserved even after the neuronal commitment was reached. FasL is a key molecule that plays a role in modulating the immune response, particularly, it is well known that stem cells expressing FasL are able to escape the immune system by inducing apoptosis in CD4^+^ and CD8^+^ T-cells (Pierdomenico et al., 2005; Zhao et al., 2012). This property is not only associated to mesenchymal stem cells but, as previously demonstrated by our group, stem cells isolated from human biliary tree - which embryologically derives from endoderm – were able to avoid the immune system through FasL expression (Riccio et al., 2014). Constitutive expression of FasL by specialized tissues, such as eye, testis and nervous system, is thought to be a key component in maintenance of immune privilege of these tissues (Green and Ferguson, 2001). It was demonstrated that neurons, as well as glial cells, are able to express FasL. The constitutive expression of FasL by neurons may limit and prevent inflammatory response and maintain relative immune suppression. As far as a potential application of stem cell therapy to the treatment of neurodegenerative diseases is concerned, not only the expression of late neuronal differentiation markers and the achievement of functional maturation, through generation of voltage-dependent currents are required, but also the maintenance of FasL is a primary requirement (Brunlid et al., 2007). The expression of FasL in the CNS has been shown to contribute to the protection of neural cells against Fas-bearing immune-reactive T-cells (Moalem et al., 1999; Medana et al., 2001), thus suggesting that FasL could be used as an immune-modulatory factor for different grafted stem cell types.

The expression of FasL by different stem cell types might therefore represent a solution to prevent cell rejection without the use of immune-suppressive treatment (Sonntag et al., 2001; Le Guern, 2003), when applied to the regeneration of different craniofacial injuries and to neurological diseases. Our data showed the ability of hDPSCs cultured in 3D sphere to maintain FasL expression even after reaching neuronal commitment, which would provide a promising tool for further investigations.

## Author Contributions

AP designed and performed most of the experiments, collected and analyzed the data, and drafted the manuscript. LB performed the *in vitro* experiments, data analysis and interpretation, and drafted the manuscript. JM and AB carried out electrophysiological analyses, acquired and interpreted data, and drafted the manuscript. MR, MO, and MLN assisted with immunofluorescence experiments, data collection, and image analysis. AdP obtained funding, assisted with data analysis, performed the statistical analysis, and supervised the experimental design. GC obtained funding, supervised the experimental design, and assisted with data analysis. All authors revised and approved the manuscript.

## Conflict of Interest Statement

The authors declare that the research was conducted in the absence of any commercial or financial relationships that could be construed as a potential conflict of interest.
